# Case of Complete Inversion of the Uterus

**Published:** 1845-12

**Authors:** E. Fisher

**Affiliations:** Waynesville, Ohio


ILLINOIS

MEDICAL & SURGICAL JOURNAL.

VOL. II. DECEMBER, 1845. NO. 9.

*Case of complete inversion of the Uterus successfully treated by* E. Fisher, M. D., of Waynesville, Ohio.

The following case, given in a private communication, is of so much interest, that I know the intelligent author of it will pardon the liberty I have taken in giving it to the public.

Complete inversion of the uterus is fortunately a rare occurrence, but it is not, as supposed by Dr. Lee, always the result of bad management. Where there is contraction of the os uteri, rendering the return of the uterus impossible, by any ordinary means, would it not be better to divide the stricture, compress the uterus to relieve its congestion, and revert it, than to abandon the woman to the horrible consequences of its remaining inverted? At an early period this might be completely successful, and it would be much less objectionable in all respects, than the removal of the whole uterus by ligature, as has been recommended, and frequently, though in most cases fatally, practiced. J. E.

Waynesville, Ohio, Nov. 18th, 1845.

Prof. Evans,

Dear Sir:—The following case of inversion of the uterus may prove of some interest to you, occupying as you do a very important chair in the Rush Medical College.

I should not probably have troubled you with it, had it not been that a medical writer of great celebrity* treats such cases as beyond the reach of remedies, abandons them to their fate, and denounces all attempts at restoration, as not only useless, but injurious—increasing their suffering without a possibility of success. That his advice would prove safe in a large proportion of such cases, I entertain no doubt; but that

* Vide Dewees' System of Midwifery, p. 479.

cases may present, in which the physician would be remiss in the discharge of duty, were he to adhere to such opinions, I think will appear plain from the following

CASE.

On the 26th of September, 1835, at ten o'clock, A. M., I was called to Mrs. D—— in labour. She was about thirty-five years of age, and had given birth to several children—said that two weeks previously, in attending the funeral of a relative, she rode several miles in a farm-wagon over rough roads, which excited pain in the loins and hips and weakness of the inferior extremities, attended with difficulty of locomotion; all of which continued up to the morning of the 26th.

From the day of the funeral she had felt no motion of the fœtus, and to use her language, was "eight months gone in pregnancy." The pains were slight, irregular, and transient. Upon examination I found the pelvis unusually large, the os uteri well dilated, and the membranes protruding. The temperature of the skin was natural, pulse regular, and bowels open.

As I conceived there was but little to fear, I deemed it prudent to give nature time to effect her purpose. X grains of pulvis doveri were administered, which procured an hour's repose. The pains then returned, but continued feeble an hour longer without any appreciable change, when suddenly a violent throe, thrust fœtus, placenta, and body of the uterus beyond the labia exteria.

The fœtus was very small and putrid, the funis umbilicalis, as nearly as I could estimate it, (not having any means of measurement,) was eight inches in length. The placenta was detached, and a complete inversion of the uterus had taken place. I was shocked for a moment with the condition of my patient, but knowing there was no time to be lost, I immediately commenced an attempt to return the uterus. I passed the index of the right hand into the vagina, then carried it round the tumor, till I became fully satisfied as to the condition of the parts. The os uteri looked into the pelvic cavity, and the finger could not be brought into contact with it.

The uterus was as flaccid as a wet bladder; a circumstance which inspired a ray of hope that something might be done to relieve the patient from a situation but little more desirable than death. I placed the fingers of my right hand against the fundus of the uterus, pressing it gently upwards in the direction of the axis of the inferior strait, while the left hand was placed over the hypogastric region to prevent the uterus from rising into the abdomen. I carried my right hand up the vagina a sufficient extent to enable me to return the uterus which was done with less difficulty than could have been anticipated. Not the slightest contraction of either the fundus, body, or neck of the organ took place during the operation. I made an attempt to withdraw my hand and the fundus followed it. And notwithstanding frictions were made over the hypogastrium with the left hand, while the knuckles of the right were caused to press against the fundus of the organ within, the uterus still remained flaccid, and during some minutes manifested no disposition to contract. I ordered ℥j of secale cornutum infused in six ounces of water, two ounces of which were to be given every ten minutes; the third portion produced contractions, and by grasping the fundus between the thumb and fingers of the left hand, through the walls of the abdomen, in less than fifteen minutes the contractions became so violent that my hand was forced out into the vagina. No further difficulty ensued. The hemorrhage was less than in an ordinary case of labour at the full period of utero-gestation, and neither pain or syncope occurred prior to or during the reversion of the organ. The practicability of returning the uterus when a complete inversion has taken place, has been doubted by Dr. Dewees, he not having been able to succeed in those cases that came under his observation, on account of the contracted condition of the cervex-uteri, and while the fundus and body remained in a relaxed condition. He conceived that, in such cases, the disproportion between the several parts of the organ was so great, and its attachment with the pelvis so low, that any attempt at restoration must prove abortive. In the case of Mrs. D., an injury had been sustained by traveling in a farm wagon over rough roads—a dead fœtus retained in the uterus some days—the funis umbilicalis some eight inches in length, and when the labour came on the fœtus appeared to be expelled almost entirely by the action of the abdominal muscles. In Dewees' cases, atony of the fundus and body existed, while the cervix and os uteri retained their contractile power. In the case under consideration the fundus, body, cervix, and os uteri were in a state of extreme atony.

